# Polymorphic Variants of the *PDGFRB* Gene Influence Efficacy of PRP Therapy in Treating Tennis Elbow: A Prospective Cohort Study

**DOI:** 10.3390/jcm11216362

**Published:** 2022-10-28

**Authors:** Karol Szyluk, Alicja Jarosz, Anna Balcerzyk-Matić, Joanna Iwanicka, Tomasz Iwanicki, Tomasz Nowak, Marcin Gierek, Marius Negru, Marcin Kalita, Sylwia Górczyńska-Kosiorz, Wojciech Kania, Paweł Niemiec

**Affiliations:** 1 Department of Physiotherapy, Faculty of Health Sciences in Katowice, Medical University of Silesia in Katowice, 40-752 Katowice, Poland; 2 District Hospital of Orthopaedics and Trauma Surgery, Bytomska 62 Str., 41-940 Piekary Śląskie, Poland; 3 Department of Biochemistry and Medical Genetics, School of Health Sciences in Katowice, Medical University of Silesia in Katowice, Medyków 18 Str., 40-752 Katowice, Poland; 4 Center for Burns Treatment, Jana Pawła II Str., 41-100 Siemianowice Śląskie, Poland; 5 Trauma and Orthopaedics Department, St. Bernard’s Hospital, Harbour Views Rd., Gibraltar GX11 1AA, UK; 6 Department of Internal Medicine, Diabetology and Nephrology, School of Medicine with the Division of Dentistry in Zabrze, Medical University of Silesia in Katowice, 41-800 Zabrze, Poland; 7 Department of Trauma and Orthopedic Surgery, Multidisciplinary Hospital in Jaworzno, Chełmońskiego 28 Str., 43-600 Jaworzno, Poland

**Keywords:** *PDGFRB*, PRP, tendinopathy, tennis elbow, SNP

## Abstract

Background: Differences in response to PRP (platelet-rich plasma) therapy may be linked to the variability of growth factors and their receptor’s genes. Considering that, we checked whether the platelet-derived growth factor receptor beta gene (*PDGFRB*) single nucleotide polymorphisms (SNPs) affect the effectiveness of PRP therapy in treating tennis elbow patients. Methods: The treatment efficacy was analyzed over time (2, 4, 8, 12, 24, 52, and 104 weeks after PRP injection) on 107 patients (132 elbows) using PROMs (patient-reported outcome measures), namely VAS (Visual Analog Scale), QDASH (quick version of Disabilities of the Arm, Shoulder, and Hand) and PRTEE (Patient-Rated Tennis Elbow Evaluation). Five polymorphisms of the *PDGFRB* gene (rs4324662, rs758588, rs3828610, rs3756311, and rs3756312) were genotyped. Results: The CC (rs3828610) and GG (rs3756311 and rs3756312) genotypes had a particularly strong impact on the effectiveness of the therapy, as measured by the values of PROMs, both in additive as well as dominant/recessive models. These homozygotes were also characterized by significantly higher values of MPV (mean platelet volume). Conclusions: The *PDGFRB* gene SNPs affect the effectiveness of PRP therapy in treating tennis elbow patients and it may result from the differentiated metabolic activity of platelets in particular genotype variants.

## 1. Introduction

Lateral epicondylitis (LE), also known as tennis elbow, is the most common cause of elbow pain and dysfunction. It can be described as an overuse syndrome of the wrist extensor muscles leading to a degeneration of the common extensor origin. The extensor carpi radialis brevis (ECRB) tendon is involved in the majority of cases, but other muscles can be involved [1].

One of the most commonly used methods for therapy is platelet-rich plasma (PRP) injection. PRP is an autologous fraction of whole blood with high concentrations of platelets [2]. The growth factors released by platelets have been shown to promote cell recruitment, proliferation, and angiogenesis, that is why it is hypothesized that PRP may promote the repair process. The use of PRP therapy is the subject of numerous discussions in the scientific community. Its effectiveness is confirmed by numerous studies, including many meta-analyses [2,3,4,5,6]. On the other hand, some authors question the evidence of its efficacy [7]. In the latest network meta-analysis of 25 randomized controlled trials with 2040 patients [8], the authors concluded that PRP therapy compared to saline control offers significant pain relief, with no significant functional benefits. The reasons for these discrepancies are undoubtedly a lack of a unified optimal preparation method and dosing of PRP, difficulties in finding an appropriate control group (even placebo injection can cause some effect and leaving the patient without any treatment would be unethical), and, importantly, the individual variability in response to this type of therapy. 

Regeneration is a complex process that is dependent on numerous exogenous and also endogenous factors, including genetic variation. It seems to be justified to search for these genetic factors amongst genes encoding growth factors, such as platelet-derived growth factor (PDGF), transforming growth factor (TGF-β), vascular endothelial growth factor (VEGF), insulin-like growth factor 1 (IGF-1), fibroblastic growth factor (FGF), and epidermal growth factor (EGF) [9]. Our previous studies identified polymorphisms within the PDGFA and PDGFB genes, which significantly influenced the concentration of platelets and the effectiveness of PRP treatment [10,11]. Looking for other genetic factors influencing an individual response to treatment, we decided to analyze platelet-derived growth factor receptor (PDGFR) genes. PDGF receptors are expressed by several cell types involved in wound healing, such as smooth muscle cells, fibroblasts, neutrophils, and macrophages. These cells are recruited to the wounded area after PDGF stimulation [12]. Based on the above, it is assumed that the polymorphisms of genes encoding receptors for growth factors may also be involved in the regeneration of musculoskeletal system injuries, including enthesopathies. Therefore, the present study aimed to check whether the *PDGFRB* gene single nucleotide polymorphisms (SNPs) affect the effectiveness of PRP therapy in treating tennis elbow patients by their effect on the patient-reported outcome measures (PROMs) values. We also investigated the possible association between *PDGFRB* gene SNPs and platelet parameters in whole blood and PRP.

## 2. Materials and Methods

This study was prepared using STROBE guidelines. The studied cohort included patients with tennis elbow, treated with PRP. The therapeutic process was analyzed over two years with follow-ups at 2, 4, 8, 12, 24, 52, and 104 weeks after the PRP injection using patient-reported outcome measures (VAS, QDASH, and PRTEE). Five polymorphisms of the *PDGFRB* gene were genotyped and treatment efficacy was compared between different genotypes. Additionally, the influence of these SNPs on platelet parameters was analyzed. 

### 2.1. Patients

Patients for this study were chosen, examined, and excluded based on the same criteria as in our previous studies on the influence of the *PDGFA* and *PDGFB* SPNs on PRP efficacy [10,11]. The studied cohort included 107 Polish Caucasians, inhabitants of Upper Silesia. There were 65 females and 42 males, aged 24–64 years, with lateral elbow tendinopathy (M77.1, according to International Statistical Classification of Diseases and Related Health Problems 10th Revision, ICD-10) treated with autologous platelet-rich plasma. Patients were treated at: the VI Department of Trauma and Orthopedics, District Hospital of Orthopedics and Trauma Surgery in Piekary Śląskie, Poland, or Department of Orthopedic Trauma Surgery, Multidisciplinary Hospital in Jaworzno, Poland. Patients were selected for the study, examined, and injected by the same orthopedic surgeons (K.S. -Piekary Śląskie and W.K.-Jaworzno), following the same study protocol.

The study included patients with typical symptoms of tennis elbow: pain in the region of the common extensor origin, pain and muscle weakness, morning stiffness, history of limb overuse and/or injury, positive Thomson’s, Mill’s tests, and Cozen’s signs, and tenderness at palpation over the lateral epicondyle of the humerus. 

**Figure 1:**
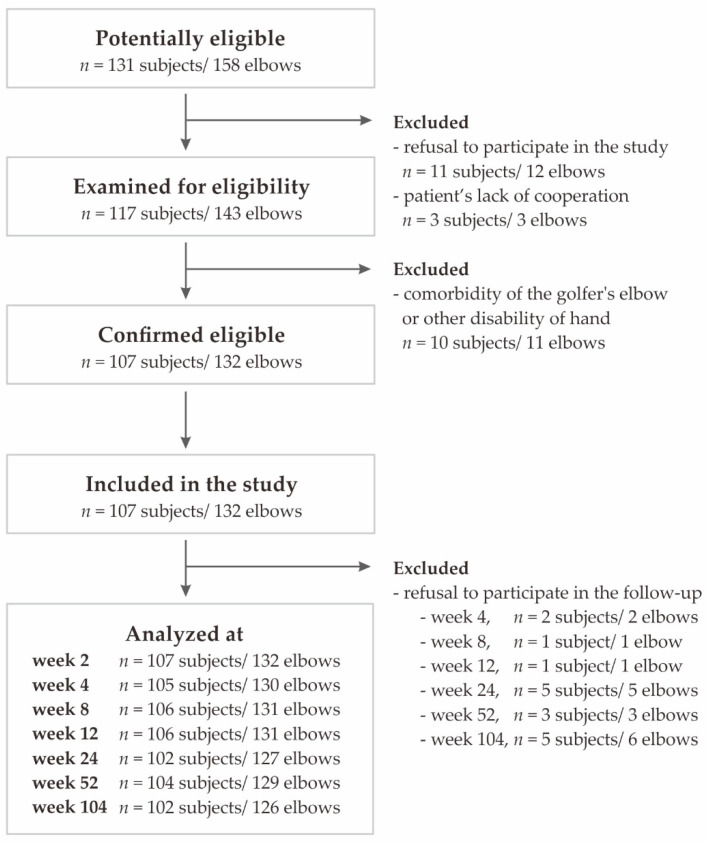
Flowchart of the study selection.

The exclusion criteria were: additional injury/disease (rheumatoid arthritis, active malignancy, cervical radiculopathy), pregnancy, prior surgical intervention, anti-platelet medication, local steroid injections in the preceding 6 months, or previous PRP injections. Patients were selected between November 2018 and November 2019. Data for the study were collected up to November 2020. There was no specific post-injection rehabilitation protocol in the present study. Additional post-injection therapy, such as steroids, nonsteroidal anti-inflammatory drugs, physiotherapy and additional injections of PRP were monitored but were not a criterion for exclusion. The flow diagram of the patients included in the study is presented in Figure 1. 

The study protocol was approved by the ethics Committee of the Medical University of Silesia in Katowice, Poland (KNW/0022/KB1/24/I/17). The methods used in this study were compliant with the Helsinki Declaration of 1975 and its further revisions. All subjects provided informed written consent. 

### 2.2. PRP Separation, Injection Procedure, Whole Blood, and PRP Parameters

PRP separation, injection procedure, and blood analysis were the same as in our previous studies [10,11]. Under standardized conditions (in a treatment room equipped with disposable equipment, 200 °C, the same exposure to light), blood was collected, separated, and PRP was injected. An Arthrex Autologous Conditioned Plasma (ACP) double syringe (Arthrex GmbH, München, Germany) was used for plasma extraction. PRP was separated from fresh whole blood immediately after blood collection. From each patient, 12 mL of whole blood was collected with the use of a 1.2 mm needle, the blood was mixed with 3.13% sodium citrate (MediPac^®^ GmbH, Königswinter, Germany) in a 9:1 ratio, and then centrifuged under the same conditions in a Rotofix 32A centrifuge (Andreas Hettich GmbH & Co, Tuttlingen, Germany). The centrifugation process was carried out at a speed of 1500 rpm and a spin time of 5 min. For patients with bilateral tennis elbow, PRP was isolated twice for each elbow separately. From each blood sample, between 2.5 and 3.5 mL of PRP was gained after centrifugation. Immediately after centrifugation, fresh PRP was as injected into the area of common extensor origin in a volume of 2.0–3.0 mL with a 1.2 mm needle. The injection was performed under ultrasound control using the Mindrinto DC-3 apparatus with a linear probe with a frequency range of 5, 7.5, and 10 MHz. The remaining 0.5 mL of PRP was saved for further analysis. 

After the PRP injection, each patient was observed for 30 min in the hospital outpatient clinic for possible complications. Particular attention was paid to local inflammation and allergic reactions. In the absence of disturbing local and general symptoms, patients were discharged. Each patient was advised to contact the hospital in case of side effects, such as a local inflammatory reaction and persistent pain. Patients were also advised to avoid heavy use of the affected hand for 24 h. No patients had an infection at the PRP injection site.

On the day of PRP injection, the complete blood count and hsCRP levels in whole blood, as well as the measures of platelets, plateletcrit (PCT), mean platelet volume (MPV), and platelet distribution width (PDW) in fresh PRP, were determined. For patients with bilateral tennis elbow, where the injections were given on the same day, a single sample of whole blood and PRP was analyzed. If the injections were carried out on different dates, two separate tests of both whole blood and PRP were performed.

### 2.3. Follow-Up, Outcomes, Measures of Effectiveness

In this study, we used the same measures of effectiveness, follow-up, and outcomes as in the previous studies [10,11]. The efficacy of PRP therapy was analyzed at 2, 4, 8, 12, 24, 52, and 104 weeks after injection. Outcome values were analyzed in comparison to the clinical condition on the day of injection (baseline value at 0 weeks). 

The VAS, QDASH, and PRTEE questionnaires were used to assess pain and disability. The VAS ranges from 0 minimum to 10 maximum pain, while QDASH and PRTEE range from 0 minimum to 100 maximum pain and disability. The effect of quantitative or qualitative variables (such as age, gender, BMI, blood count, PRP parameters, *PDGFRB* genotypes, etc.) on treatment efficacy was determined from raw VAS, QDASH, and PRTEE values and compared to baseline values (ΔVAS, ΔQDASH, and ΔPRTEE). If there were statistically significant differences in raw PROMs values at the start of the study, only the results of ΔVAS, ΔQDASH, and/or ΔPRTEE were taken into account.

### 2.4. Genetic Analyses

The genomic DNA was isolated from peripheral blood leukocytes using the MasterPure genomic DNA purification kit (Epicenter Technologies, Madison, WI, USA). SNPs of the *PDGFRB* gene were genotyped using the TaqMan Predesigned SNP Genotyping Assay kits (Thermo Fisher Scientific, Waltham, MASS, USA) and the 7300 Real-Time PCR System (Thermo Fisher Scientific, Waltham, MASS, USA). The accuracy of genotyping was checked by regenotyping 10–15% of the samples. The repeatability of the results was 100%.

**Figure 2:**
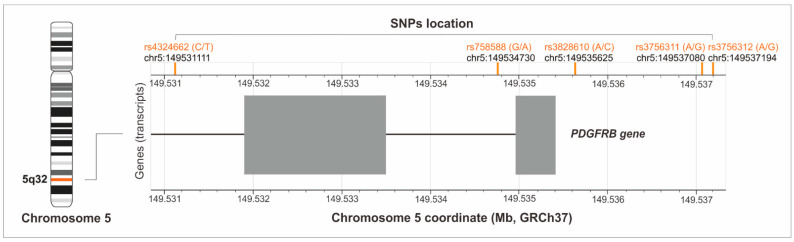
Location of the studied polymorphisms on chromosome 5 (the figure was created with the use of LDmatrix Tool [14]).

Only SNPs with a minor allele frequency (MAF) ≥ 20% in populations of European origin, based on the Database of SNPs of the National Center for Biotechnology Information, U.S. National Library of Medicine [13], were selected for analysis. There were rs4324662 (C > T), rs758588 (G > A), rs3828610 (A > C), rs3756311 (A > G), and rs3756312 (A > G) variants. The first two are intronic polymorphisms, the other three are located in the 5′-upstream promoter regions of the *PDGFRB* gene (Figure 2).

### 2.5. Statistical Analyses

Statistical analysis was performed using Statistica 13.0 software (TIBCO Software Inc, Palo Alto, CA, USA). The Shapiro–Wilk test was used to check the normality of the distribution. As the quantitative variables showed non-normal distribution, data were compared using the Mann–Whitney U test, and Kruskal–Wallis test. The results of quantitative data were reported as the median and their spread as a quartile deviation (QD). The Spearman’s rank correlation coefficient, the r_s_, was used as a measure of the correlation between quantitative variables. Cases with missing data were rejected from the respective comparisons. 

Genetic data were analyzed in additive and dominant/recessive inheritance models. The Hardy–Weinberg equilibrium was tested with the χ^2^ test, as well as comparisons of genotype and allele frequencies. The Fisher’s correction was applied in subgroups with less than ten patients. Haplotype blocks were defined using the HaploView software [15] using Gabriel et al. algorithm [16]. The values of D‘ and r^2^ were used as linkage disequilibrium measures. The study size and power analysis were computed using a sample size *t* test-median and SD tool, available on https://rbiostatistics.com/sample_size_t_test_median (accessed on 19 October 2022) [17]. The power of all statistically significant tests in the current work was greater than 85% with a 95% two-sided confidence level. Statistical significance was accepted at *p* < 0.050. In the case of multiple comparisons, the *p* values were adjusted using the Bonferroni correction.

## 3. Results

### 3.1. General Characteristics of the Study Group

The study population included 107 patients, the majority of whom were women (58.3% vs. 41.7%). The median (±QD) age was 46.00 ± 5.50 years and the median BMI was 25.65 ± 2.00. A total of 132 elbows were treated, 65.2% of which were on the dominant side. The most common comorbidities were hypertension (13.6%), thyroid disease (11.4%), and gout (6.1%). The median platelets concentration (PLT) in the whole blood was 240.00 ± 40.50 (10^9^/L ± QD). The number of platelets was greater in women than in men (261.50 ± 33.00 vs. 224.00 ± 38.75, respectively, *p* = 0.001). The median platelets volume (MPV) in the whole blood was 9.10 ± 0.73 (fl ± QD) and there was no statistical difference between the sexes (*p* > 0.050). The median plateletcrit (PCT) in the whole blood was 2.31 ± 0.36 (mL/L ± QD) and was higher in women than in men (2.37 ± 0.36 vs. 2.04 ± 0.33, respectively, *p* = 0.001). Table 1 provides summarized demographic, clinical, and biochemical data for the study population.

### 3.2. Analysis of the PDGFRB Gene Polymorphisms

Genotyping data were obtained for 107 subjects (132 elbows). Genotype frequencies of four of the five SNPs were compatible with the Hardy–Weinberg equilibrium (*p* > 0.050). The only exception was the rs758588 polymorphism (*p* = 0.030). The frequencies of genotypes and alleles of the analyzed *PDGFRB* gene polymorphisms are presented in Table 2. 

The studied polymorphisms create two haplotypes. The first one includes rs4324662 and rs758588 SNPs (r^2^ = 0.81, D’ = 1.00). The second haplotype includes rs3828610, rs3756311, and rs3756312 polymorphisms (r^2^ = 1.00, D’ = 1.00 for rs3828610 and rs3756311; r^2^ = 0.69, D’ = 1.00 for rs3756311 and rs3756312 and r^2^ = 0.69, D’ = 1.00 for rs3828610 and rs3756312 SNPs). 

### 3.3. Polymorphisms of the PDGFRB Gene and Clinical Phenotype

**Figure 3:**
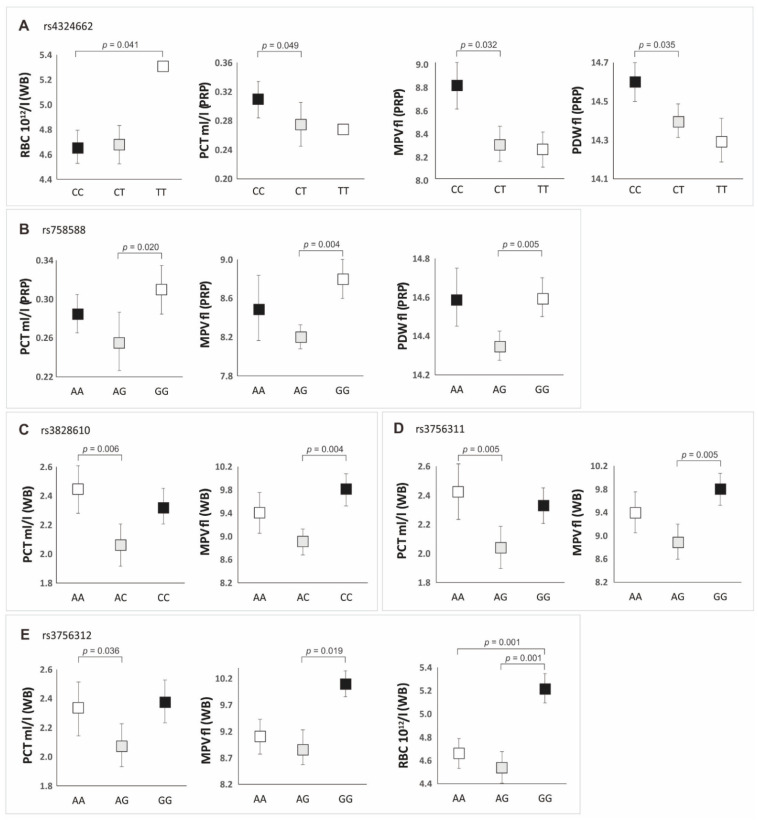
Whole blood (WB) and platelet-rich plasma (PRP) parameter values in individuals with particular genotypes of *PDGFRB* gene polymorphisms (additive model): (**A**) for rs4324662; (**B**) for rs758588; (**C**) for rs3828610; (**D**) for rs3756311; (**E**) for rs3756312 SNP.

We have analyzed the basic clinical and demographic parameters of patients depending on the genotypes of the polymorphisms studied (Table S1). The analysis of whole blood and PRP parameters in the context of *PDGFRB* gene variants was also made, both in the additive and dominant/recessive models (Tables S2 and S3, Figure 3). 

The age of the respondents, their BMI, the frequency of risk factors, and their comorbidities did not differentiate patients with particular genotypes (Table S1). The same applied to the use of additional forms of therapy during follow-ups. The exceptions were the observed difference in the frequency of hypertension between subjects with the CC and TT genotypes (rs4324662 polymorphism) and the frequency of physical therapy between the AG and GG genotypes (rs758588 polymorphism) (Table S1). In both cases, these genotypes did not influence the effectiveness of PRP therapy, which was discussed later in the study. 

The factor that significantly differentiated particular genotypes (*p* < 0.050) was, however, the number of alcohol units consumed per week (Table S1). The most alcohol units were consumed by the cases with the genotype TT (rs4324662), AA (rs758588), CC (rs3828610), and GG (rs3756311 and rs3756312) (Table S1). In most cases, these patients had lower levels of PLT (in whole blood and PRP), compared to other genotypes of individual polymorphisms (Table S2). It should be noted, however, that the differences in the concentration of platelets between individual genotypes were not statistically significant (Table S2). Nevertheless, we observed a weak negative correlation, independent of *PDGFRB* genotypes, between the number of alcohol units consumed per week and the number of platelets in whole blood (r_s_ = −0.31, *p* < 0.050). The remaining platelet parameters that significantly differentiated the genotypes of the studied SNPs in the additive model are presented in Figure 3. A summary of the dominant/recessive model is presented in Table 3.

In the additive model, the CC genotype of the rs4324662 polymorphism was characterized by higher values of PCT, MPV, and PDW in the PRP preparation compared to the other genotypes. In the case of the rs758588 polymorphism, the highest values of PCT, MPV, and PDW in PRP were found in GG homozygotes. Increased blood PCT values were related to the AA genotypes of rs3828610 and rs3756311 polymorphisms and the CC (rs3828610) and GG (rs3756311) genotypes had the highest MPV values compared to the other genotypes. The GG genotype of the rs3756312 polymorphism was characterized by the highest values of PCT, MPV, and RBC in whole blood (Figure 3, Table S2).

In the dominant/recessive model, the CC homozygotes of the rs4324662 polymorphism had higher PLT, PCT, MPV, and PDW values in the PRP preparation compared to the carriers of the T allele. Additionally, GG genotypes of the rs3756311/rs3756312 and CC genotype of the rs3828610 were characterized by higher MPV and RBC values in whole blood compared to the carriers of the A allele (Table 3 and Table S3).

**Figure 4:**
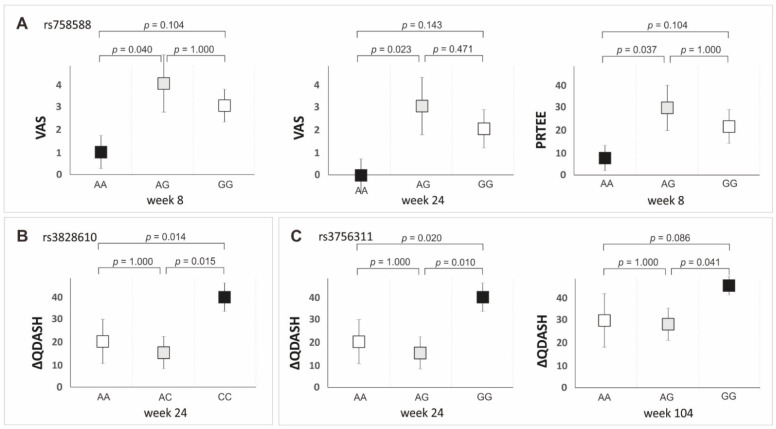
Medians (±QD) of PROMs values for genotype variants of the *PDGFRB* gene polymorphisms in the additive model: (**A**) VAS, weeks 8 and 24, PRTEE week 8 for rs758588; (**B**) ΔQDASH, week 24 for rs3828610; (**C**) ΔQDASH, weeks 24 and 104 for rs3756311 SNP.

Next, we compared the effectiveness of PRP treatment of tennis elbow by analyzing the PROM values between the different genotypes of the tested SNPs (additive model) and allelic variants (dominant/recessive model). The influence of the studied polymorphisms on the effectiveness of PRP therapy was shown in the additive model for rs758588, rs3828610, and rs3756311 SNPs (Figure 4, Table S4). The AA homozygotes of the rs758588 SNP had a significantly lower VAS (week 8 and 24) and PRTEE (week 8) than the AG heterozygotes. The CC homozygotes of the rs3828610 polymorphism had a higher ∆QDASH value at week 24 compared to the AA homozygotes and AG heterozygotes. In the case of the rs3756311 variant, higher values of ∆QDASH were observed at 24 and 104 weeks in the GG homozygotes compared to the AA homozygotes and AG heterozygotes. There were no statistically significant differences between AA and AC (rs3828610) as well as AA and AG (rs3756311) genotypes (Figure 4).

**Figure 5:**
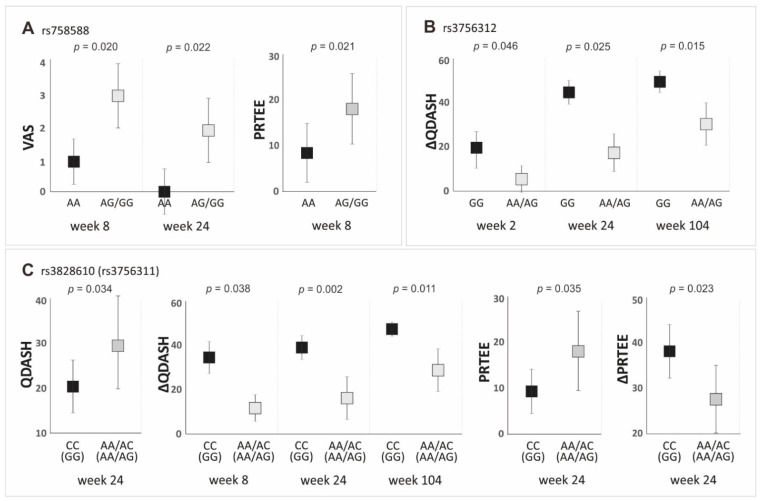
Medians (±QD) of PROMs values with respect to genotype variants of the *PDGFRB* gene polymorphisms in the dominant/recessive model: (**A**) for VAS (weeks 8 and 24) and PRTEE (week 8), for rs758588 SNP; (**B**) for ΔQDASH (weeks 2, 24, and 104), for rs3756312 SNP; (**C**) for QDASH (week 24), ΔQDASH (weeks 8, 24, and 104), PRTEE (week 24) and ΔPRTEE (week 24), for rs3828610 and rs3756311 SNPs.

Statistically significant results (*p* < 0.050) from the analysis of the effect of *PDGFRB* gene variants on PROMs values in the dominant/recessive model are presented in Figure 5. The detailed data (median ± QD, *p*-value) for each of the studied polymorphisms are presented in supplementary tables (Tables S5–S9). 

Regarding the rs4324662 polymorphism, we did not find statistically significant differences between variants for the analyzed PROMs in the dominant/recessive model (Table S5). This confirms the results obtained in the additive model. Variants of the next polymorphism of the first haplotype, namely rs758588, differentiate the values of PROMs (VAS and PRTEE) at the same follow-up time points as in the additive model. The AA homozygotes of the rs758588 polymorphism had lower VAS values at weeks 8 and 24, as well as lower PRTEE values at week 8, relative to G allele carriers (Figure 5A, Table S6). In the recessive/dominant model, lower values of QDASH (week 24) and PRTEE (week 24) and higher values of ∆QDASH (weeks 8, 24, and 104) and ∆PRTEE (week 24) were observed in the CC homozygotes of the rs3828610 than in the A allele carriers (Figure 5C, Table S7). The same differences concerned the completely linked rs3756311 polymorphism, with GG homozygotes having more favorable PROMs values than the carriers of the A allele (Figure 5C, Table S8). The GG homozygotes of the rs3756312 polymorphism also had higher values of ∆QDASH (weeks 2, 24, and 104) than the A allele carriers (Figure 5B, Table S9). 

## 4. Discussion

In the present study, we have shown that *PDGFRB* gene polymorphic variants affect the effectiveness of PRP treatment of tennis elbow. The AA (rs758588), CC (rs3828610), and GG (rs3756311 and rs3756312) genotypes had a particularly strong impact on the effectiveness of the therapy, as measured by the values of PROMs (VAS, QDASH, and PRTEE). These homozygotes responded better to the treatment than the carriers of the other genotypes. The SNPs of the *PDGFRB* gene that were also characterized by a differentiated platelet parameters profile and genotypes associated with better effectiveness of therapy had higher values of MPV. 

Importantly, the only factor influencing the treatment efficacy in this study was the genotype. BMI, smoking, alcohol consumption, or comorbidities did not affect the pain parameters. Even factors such as age and sex, previously associated with the effectiveness of therapy [18,19], did not affect it in our study.

The *PDGFRB* gene encodes the PDGFRB receptor that is involved in the development of blood vessels and the healing process [12]. According to studies in mice, PDGFRB signaling is required for the activation and recruitment of SSPCs (skeletal stem, and progenitor cells) during bone repair. Furthermore, in *Pdgfb* overexpressing mice, PDGF-B, and PDGFRB signaling have been shown to control key aspects of both osteogenesis, and angiogenesis in the postnatal bone [20]. It may be supposed that the polymorphisms of genes encoding growth factors or their receptors may affect the effectiveness of PRP treatment due to the participation of these factors in regenerative processes [9]. In our previous studies, we reported that the SNPs of the *PDGFA* and *PDGFB* genes may affect the effectiveness of PRP therapy in the treatment of tennis elbow [10,11]. Combining these results with the outcomes of the present study, it can be concluded that the presence of certain SNPs influences the individual response of patients to PRP therapy. 

Unfortunately, the literature lacks other studies related to the influence of polymorphisms of mentioned genes on treatment with PRP or injuries and the regeneration of the musculoskeletal system. There is also little evidence on the relationship between the studied polymorphisms and the expression of the *PDGFRB* gene. An exception is a work by Kim et al. [21] on the genetic association between SNPs of the *PDGFRB* gene and schizophrenia. In addition to the case–control analysis of six *PDGFRB* gene polymorphisms (including rs3756311 and rs3756312 analyzed in the present work), the authors also studied the functional effect of the identified SNPs and haplotypes on promoter activity performing luciferase activity assay in human SH-SY5Y neuroblastoma cells. Significantly enhanced luciferase activity was detected in cells transfected with the plasmid construct containing two G alleles, both of rs3756311 and rs3756312 polymorphisms. The results of this study may explain the reason why GG homozygotes within the rs3756312 and rs3756311 polymorphisms (as well as the CC homozygotes of the rs3828610, completely linked with rs3756311 SNP) were found to have a greater therapeutic efficacy of PRP in the current study. The observed increased effectiveness of the therapy may result from the increased activity of the promoter of the *PDGFRB* gene in these homozygotes. Increased promoter activity is in turn associated with increased gene expression and consequently stronger cell signaling dependent on PDGFRB.

Platelets are considered to be responsible for tissue regeneration during PRP therapy [2]. Surprisingly, genotypes associated with more effective treatment in our study (CC for rs3828610, GG for rs3756311, and GG for rs3756312 SNPs) were characterized by lower PLT levels. It should be pointed out, however, that the differences in the PLT concentration between respective genotypes were not statistically significant (*p* > 0.050). Subjects with these genotypes, which responded better to PRP therapy, had lower PLT but significantly higher MPV than the carriers of the other genotypes. According to numerous studies, a higher MPV value is associated with greater platelet activity [22,23,24,25]. Therefore, it can be assumed that the presence of larger, and therefore more active, platelets could have a beneficial effect on the effectiveness of PRP in these homozygotes.

We suppose that the effect of the *PDGFRB* gene SNPs on PRP effectiveness should be similar in other tendon injuries. However, the obtained results do not allow us to conclude that the studied polymorphisms may affect the treatment progress in other types of tissues of the musculoskeletal system. Based on in silico analyzes, it can be concluded that the PDGFRB is active in muscles, fibroblasts, and chondrocytes [26,27]. Moreover, as we noted above, *PDGFRB* gene and PDGFRB signaling may also be involved in bone regeneration [20]. However, in order to determine the exact effect of *PDGFRB* gene polymorphisms on the PRP treatment of injuries in other tissues of the musculoskeletal system, further and additional studies are necessary.

The limiting factors of our study are the relatively small size of the study group and the lack of a formal rehabilitation protocol after PRP therapy. The lack of standardization of the rehabilitation protocol was dictated by professional ethics. According to it, denying patients (with different responses to PRP therapies) access to other forms of therapy was not possible. 

## 5. Conclusions

In conclusion, the difference in response to PRP injections in tennis elbow treatment may be due to the polymorphic variability of the *PDGFRB* gene. Better efficacy of PRP therapy in CC (rs3828610) and GG (rs3756311 and rs3756312) homozygotes may result from the higher metabolic activity of platelets (higher MPV values than in other genotypes), as well as higher *PDGFRB* gene promoter activity in these individuals. The results of this study may contribute to the identification of genetic markers for PRP treatment efficacy that may find clinical application in the future. Genotyping two of these three polymorphisms of the *PDGFRB* gene (rs3828610 or rs3756311 and rs3756312) may serve as a helpful diagnostic tool in selecting therapies for treating tennis elbow. However, due to the limitations of this work, further functional and clinical studies should be carried out to verify the obtained results and expand the knowledge about the studied polymorphisms.

## Figures and Tables

**Table 1:** Demographic, clinical, and biochemical characteristics of the study group at baseline (baseline week 0).

Characteristics			
**General**	number of subjects, N	107	-
	number of elbows, n (%)	132	(100.0)
	tennis elbow in the dominant hand, n (%)	86	(65.2)
	tennis elbow in the non-dominant hand, n (%)	46	(34.8)
	females, n (%)	77	(58.3)
	age, median ± QD	46.00	5.50
	BMI, median ± QD	25.65	2.00
	overweight/obesity, BMI ≥ 25, n (%)	86	(65.2)
	current smokers, n (%)	22	(16.6)
	former smokers, n (%)	48	(36.4)
**Comorbidities**	diabetes mellitus, n (%)	4	(3.0)
	gout, n (%)	8	(6.1)
	thyroid diseases, n (%)	15	(11.4)
	hypercholesterolemia, n (%)	7	(5.3)
	hypertension, n (%)	18	(13.6)
	heart failure, n (%)	4	(3.0)
**Whole Blood**	PLT 10^9^/L, median ± QD	240.00	40.50
**parameters**	PCT mL/L, median ± QD	2.31	0.36
	MPV fl, median ± QD	9.10	0.73
	PDW fl, median ± QD	16.10	0.15
	WBC 10^9^/L, median ± QD	6.26	1.16
	RBC 10^12^/L, median ± QD	4.66	0.29
**PRP parameters**	PLT 10^9^/L, median ± QD	343.00	65.00
	PCT ml/L, median ± QD	0.30	0.06
	MPV fl, median ± QD	8.60	0.40
	PDW fl, median ± QD	14.60	0.25

Legend: BMI, body mass index; MPV, platelet volume; PCT, plateletcrit; PDW, platelet distribution width; PLT, platelets; PRP, platelet-rich plasma; RBC, red blood cells; QD, Quartile Deviation; WB, whole blood; WBC, white blood cells.

**Table 2:** The frequencies of genotypes, and alleles of analyzed SNPs of the *PDGFRB* gene.

SNP	Genotypes	n (%)	Alleles	n (%)
**rs4324662**	CC	88 (66.7)	C	216 (81.8)
	CT	40 (30.3)	T	48 (18.2)
	TT	4 (3.0)		
	CC + CT	128 (96.9)		
	TT + CT	44 (33.3)		
**rs758588**	AA	11 (8.3)	A	56 (21.2)
	AG	34 (25.8)	G	208 (78.8)
	GG	87 (65.9)		
	AA + AG	45 (34.1)		
	AG + GG	121 (91.7)		
**rs3828610**	AA	56 (42.4)	A	163 (61.7)
	AC	51 (38.6)	C	101 (38.3)
	CC	25 (18.9)		
	AA + AC	107 (81.1)		
	CC + AC	76 (57.6)		
**rs3756311**	AA	55 (41.7)	A	162 (61.4)
	AG	52 (39.4)	G	102 (38.6)
	GG	25 (18.9)		
	AA + AG	107 (81.1)		
	GG + AG	77 (58.3)		
**rs3756312**	AA	64 (48.5)	A	180 (68.2)
	AG	52 (39.4)	G	84 (31.8)
	GG	16 (12.1)		
	AA + AG	116 (87.9)		
	GG + AG	68 (51.5)		

Legend: PDGFRB, platelet-derived growth factor beta receptor gene; SNP, single nucleotide polymorphism.

**Table 3:** Whole blood (WB) and platelet-rich plasma (PRP) parameter values with respect to the *PDGFRB* gene polymorphisms variants (dominant/recessive model).

Rs Number	Parameter (Source)	Median	±QD	Median	±QD	*p* Mann–Whitney U Test
rs4324662		CC		CT/CT		
	PLT 10^9^/L (PRP)	353.00	70.50	327.00	59.75	0.024
	PCT mL/L (PRP)	0.31	0.05	0.27	0.05	0.009
	MPV fl (PRP)	8.80	0.40	8.30	0.32	0.006
	PDW fl (PRP)	14.60	0.20	14.40	0.18	0.005
rs3828610		CC		AA/AC		
	MPV fl (WB)	9.80	0.55	9.00	0.70	0.003
	RBC 10^12^/L (WB)	4.96	0.33	4.66	0.25	0.024
rs3756311		GG		AA/AG		
	MPV fl (WB)	9.80	0.55	9.00	0.70	0.003
	RBC 10^12^/L (WB)	4.96	0.33	4.66	0.25	0.024
rs3756312		GG		AA/AG		
	MPV fl (WB)	10.10	0.50	9.00	0.70	0.005
	RBC 10^12^/L (WB)	5.22	0.26	4.65	0.26	0.000

Legend: MPV, mean platelet volume; PCT, plateletcrit; PDGFRB, platelet-derived growth factor receptor beta gene; PLT, platelets; PRP, platelet-rich plasma; QD, Quartile Deviation; WB, whole blood.

## Data Availability

Not applicable.

## Supplementary Materials

The following supporting information can be downloaded at: https://www.mdpi.com/article/10.3390/jcm11216362/s1. Table S1: The frequencies (%) and medians (±QD) of basic demographic and clinical characteristics of patients concerning genotypes of the *PDGFRB* gene polymorphisms; Table S2: Whole blood (WB) and platelet-rich plasma (PRP) parameter values in individuals with particular genotypes of the *PDGFRB* gene polymorphisms (additive model); Table S3: Whole blood (WB) and platelet-rich plasma (PRP) parameters values in individuals with particular variants of the *PDGFRB* gene polymorphisms in recessive/dominant models; Table S4: PROMs values in individuals with particular genotypes of the *PDGFRB* gene polymorphisms in the additive model.; Table S5: PROMs values in TT homozygotes and carriers of the C allele of the rs4324662 *PDGFRB* gene polymorphism; Table S6: PROMs values in AA homozygotes and carriers of the G allele of the rs758588 *PDGFRB* gene polymorphism; Table S7: PROMs values in CC homozygotes and A allele carriers of the rs3828610 *PDGFRB* gene polymorphism; Table S8: PROMs values in GG homozygotes and A allele carriers of the rs3756311 *PDGFRB* gene polymorphism; Table S9: PROMs values in GG homozygotes and A allele carriers of the rs3756312 *PDGFRB* gene polymorphism.
