# Supplementary material for: Polymorphic Variants of the PDGFRB Gene Influence Efficacy of PRP Therapy in Treating Tennis Elbow: A Prospective Cohort Study
Source: J Clin Med. 2022 Oct 28;11(21):6362. doi: 10.3390/jcm11216362 (PMC9657684; doi:10.3390/jcm11216362)
Supplement: Supplementary file 1 [file jcm-11-06362-s001.zip › Table S1.pdf]

Table S1. The frequencies (%) and medians (± QD) of basic demographic and clinical characteristics of patients in relation to genotypes of the *PDGFRB* gene polymorphisms.

**Parameter**	**Genotypes of rs4324662**						***P* value**			
	**CC**		**CT**		**TT**					
	**Median or *n***	**±QD or %**	**Median or *n***	**±QD or %**	**Median or *n***	**±QD or %**	**Kruskal-Wallis test**	**CC vs CT**	**CC vs TT**	**CT vs TT**
Age years, median ± QD	46.50	5.00	45.00	6.25	42.00	6.25	0.422			
BMI, median ± QD	25.99	2.42	25.08	2.13	24.44	1.24	0.545			
Cigarettes n/day, median ± QD	0.00	0.00	0.00	0.00	10.00	10.00	0.051			
Alcohol units/ week, median ± QD	0.00	1.00	2.00	2.00	4.50	1.75	**0.000**	**0.005**	**0.031**	0.528
Male sex, n (%)	35	39.77	17	42.50	3	75.00	-			
Cigarette smoking, n (%)	17	19.32	3	7.50	2	50.00	-			
Hypertension, n (%)	16	18.18	2	5.00	0	0.00	-	**0.048**		
Physical therapy, n (%)	33	37.50	20	50.00	3	75.00	-			
Manual therapy, n (%)	27	30.68	7	17.50	3	75.00	-			
NSAIDs, n (%)	28	31.82	11	27.50	2	50.00	-			
**Parameter**	**Genotypes of rs758588**						***P* value**			
	**AA**		**AG**		**GG**					
	**Median or *n***	**±QD or %**	**Median or *n***	**±QD or %**	**Median or *n***	**±QD or %**	**Kruskal-Wallis test**	**AA vs AG**	**AA vs GG**	**AG vs GG**
Age years, median ± QD	49.00	9.00	44.50	4.50	47.00	5.00	0.263			
BMI, median ± QD	24.86	1.83	25.13	2.13	25.95	2.37	0.773			
Cigarettes n/day, median ± QD	0.00	10.00	0.00	0.00	0.00	0.00	0.104			
Alcohol units/ week, median ± QD	3.00	2.00	2.00	2.00	0.00	1.00	**0.001**	0.893	**0.015**	**0.024**
Male sex, n (%)	8	72.73	12	35.29	35	40.23	-			
Cigarette smoking, n (%)	3	27.27	2	5.88	17	19.54	-			
Hypertension, n (%)	0	0.00	2	5.88	16	18.39	-			
Physical therapy, n (%)	4	36.36	20	58.82	32	36.78	-			**0.028**
Manual therapy, n (%)	3	27.27	8	23.53	26	29.89	-			
NSAIDs, n (%)	5	45.45	8	23.53	28	32.18	-			
**Parameter**	**Genotypes of rs3828610**						***P* value**			
	**AA**		**AC**		**CC**					

	Median or *n*	±QD or %	Median or *n*	±QD or %	Median or *n*	±QD or %	Kruskal-Wallis test	AA vs AC	AA vs CC	AC vs CC
Age years, median ± QD	47.00	6.00	45.00	4.50	45.00	5.00	0.630			
BMI, median ± QD	25.86	1.79	25.28	2.65	25.83	2.64	0.962			
Cigarettes n/day, median ± QD	0.00	0.00	0.00	0.00	0.00	0.00	0.848			
Alcohol units/ week, median ± QD	0.00	1.00	1.00	2.00	2.00	2.00	**0.005**	**0.037**	**0.027**	1.000
Male sex, n (%)	20	35.71	23	45.10	12	48.00	-			
Cigarette smoking, n (%)	9	16.07	10	19.61	3	12.00	-			
Hypertension, n (%)	10	17.86	6	11.76	2	8.00	-			
Physical therapy, n (%)	22	39.29	21	41.18	13	52.00	-			
Manual therapy, n (%)	15	26.79	15	29.41	7	28.00	-			
NSAIDs, n (%)	20	35.71	15	29.41	6	24.00	-			

**Parameter**	**Genotypes of rs3756311**						***P* value**			
	**AA**		**AG**		**GG**					
	**Median or *n***	**±QD or %**	**Median or *n***	**±QD or %**	**Median or *n***	**±QD or %**	**Kruskal-Wallis test**	**AA vs AG**	**AA vs GG**	**AG vs GG**
Age years, median ± QD	47.00	5.00	45.00	4.50	45.00	5.00	0.449			
BMI, median ± QD	25.88	1.67	25.23	2.92	25.83	2.64	0.890			
Cigarettes n/day, median ± QD	0.00	0.00	0.00	0.00	0.00	0.00	0.703			
Alcohol units/ week, median ± QD	0.00	1.00	1.00	2.00	2.00	2.00	**0.005**	**0.033**	**0.025**	1.000
Male sex, n (%)	20	36.36	23	44.23	12	48.00	-			
Cigarette smoking, n (%)	8	14.54	11	21.15	3	12.00	-			
Hypertension, n (%)	10	18.18	6	11.54	2	8.00	-			
Physical therapy, n (%)	21	38.18	22	42.31	13	52.00	-			
Manual therapy, n (%)	15	27.27	15	28.85	7	28.00	-			
NSAIDs, n (%)	19	34.54	16	30.77	6	24.00	-			

**Parameter**	**Genotypes of rs3756312**						***P* value**			
	**AA**		**AG**		**GG**					
	**Median or *n***	**±QD or %**	**Median or *n***	**±QD or %**	**Median or *n***	**±QD or %**	**Kruskal-Wallis test**	**AA vs AG**	**AA vs GG**	**AG vs GG**
Age years, median ± QD	47.00	5.50	45.00	5.50	44.50	4.00	0.377			
BMI, median ± QD	26.23	2.33	24.99	2.13	25.83	1.99	0.539			
Cigarettes n/day, median ± QD	0.00	0.00	0.00	0.00	0.00	0.00	0.935			
Alcohol units/ week, median ± QD	0.00	1.00	1.00	2.00	2.00	1.50	**0.015**	0.222	**0.037**	0.614

Male sex, n (%)	24	37.50	20	38.46	11	68.75	-	**0.049**
Cigarette smoking, n (%)	11	17.19	9	17.31	2	12.50	-	
Hypertension, n (%)	12	18.75	6	11.54	0	0.00	-	
Physical therapy, n (%)	25	39.06	23	44.23	8	50.00	-	
Manual therapy, n (%)	20	31.25	12	23.08	5	31.25	-	
NSAIDs, n (%)	21	32.81	17	32.69	3	18.75	-	

Legend: BMI, body mass index; NSAIDs, nonsteroidal anti-inflammatory drugs; QD, quartile deviation.
