# Supplementary material for: Polymorphic Variants of the PDGFRB Gene Influence Efficacy of PRP Therapy in Treating Tennis Elbow: A Prospective Cohort Study
Source: J Clin Med. 2022 Oct 28;11(21):6362. doi: 10.3390/jcm11216362 (PMC9657684; doi:10.3390/jcm11216362)
Supplement: Supplementary file 1 [file jcm-11-06362-s001.zip › Table S2.pdf]

Table S2. Whole blood (WB) and platelet-rich plasma (PRP) parameters values in individuals with particular genotypes of the *PDGFRB* gene polymorphisms (additive model).

Parameter	Source	Genotypes of rs4324662						*P* value			
		CC		CT		TT					
		Median	±QD	Median	±QD	Median	±QD	Kruskal-Wallis test	CC vs CT	CC vs TT	CT vs TT
PLT 10^9/l	WB	246.00	39.00	235.00	39.25	238.00	30.00	0.418			
PCT ml/l	WB	2.28	0.39	2.35	0.32	2.33	0.32	0.638			
MPV fl	WB	9.10	0.70	9.00	0.95	9.80	0.15	0.564			
PDW fl	WB	16.10	0.15	16.10	0.15	16.00	0.10	0.958			
WBC 10^9/l	WB	6.24	1.16	6.19	0.99	9.19	2.14	0.520			
RBC 10^{12}/l	WB	4.66	0.27	4.68	0.31	5.30	0.01	0.048	1.000	0.041	0.062
PLT 10^9/l	PRP	353.00	70.50	312.00	67.25	331.50	16.50	0.076			
PCT ml/l	PRP	0.31	0.05	0.28	0.06	0.27	0.01	0.031	0.049	0.502	1.000
MPV fl	PRP	8.80	0.40	8.30	0.30	8.25	0.30	0.019	0.032	0.421	1.000
PDW fl	PRP	14.60	0.20	14.40	0.17	14.30	0.23	0.015	0.035	0.257	1.000

Parameter	Source	Genotypes of rs758588						*P* value			
		AA		AG		GG					
		Median	±QD	Median	±QD	Median	±QD	Kruskal-Wallis test	AA vs AG	AA vs GG	AG vs GG
PLT 10^9/l	WB	221.00	43.50	244.00	28.50	246.00	39.00	0.424			
PCT ml/l	WB	2.13	0.22	2.38	0.34	2.25	0.39	0.490			
MPV fl	WB	9.65	0.85	9.00	0.95	9.10	0.70	0.910			
PDW fl	WB	16.15	0.10	16.00	0.15	16.10	0.15	0.472			
WBC 10^9/l	WB	6.60	1.97	6.19	1.13	6.24	1.16	0.838			
RBC 10^{12}/l	WB	4.63	0.48	4.71	0.33	4.66	0.27	0.796			
PLT 10^9/l	PRP	335.00	44.00	307.00	64.00	353.00	70.50	0.067			
PCT ml/l	PRP	0.29	0.04	0.26	0.06	0.31	0.05	0.022	1.000	0.955	0.020
MPV fl	PRP	8.50	0.68	8.20	0.25	8.80	0.40	0.006	0.557	1.000	0.004
PDW fl	PRP	14.60	0.30	14.35	0.15	14.60	0.20	0.007	0.573	1.000	0.005

Parameter	Source	Genotypes of rs3828610						*P* value			
		AA		AC		CC					
		Median	±QD	Median	±QD	Median	±QD	Kruskal-Wallis test	AA vs AC	AA vs CC	AC vs CC
PLT 10^9/l	WB	256.00	37.50	226.00	35.00	228.00	40.50	0.055			
PCT ml/l	WB	2.46	0.34	2.06	0.29	2.33	0.25	0.009	0.006	0.603	0.684
MPV fl	WB	9.40	0.70	8.90	0.45	9.80	0.55	0.006	0.556	0.084	0.004

PDW fl	WB	16.00	0.20	16.10	0.10	16.20	0.15	0.113			
WBC 10^9/l	WB	6.14	1.14	6.24	1.04	6.70	1.13	0.896			
RBC 10^{12}/l	WB	4.61	0.27	4.67	0.21	4.96	0.33	0.064			
PLT 10^9/l	PRP	351.00	90.75	341.00	65.00	328.00	44.50	0.202			
PCT ml/l	PRP	0.31	0.07	0.28	0.07	0.28	0.04	0.177			
MPV fl	PRP	8.70	0.48	8.50	0.40	8.50	0.50	0.409			
PDW fl	PRP	14.60	0.20	14.60	0.25	14.50	0.20	0.727			

**Parameter**	**Source**	**Genotypes of rs3756311**						**P value**			
		**AA**		**AG**		**GG**					
		**Median**	**±QD**	**Median**	**±QD**	**Median**	**±QD**	**Kruskal-Wallis test**	**AA vs AG**	**AA vs GG**	**AG vs GG**
PLT 10^9/l	WB	257.00	37.50	226.00	35.00	228.00	40.50	0.035	0.070	0.129	1.000
PCT ml/l	WB	2.46	0.34	2.04	0.29	2.33	0.25	0.007	0.005	0.541	0.666
MPV fl	WB	9.40	0.70	8.90	0.60	9.80	0.55	0.007	0.790	0.072	0.005
PDW fl	WB	16.00	0.20	16.10	0.10	16.20	0.15	0.122			
WBC 10^9/l	WB	6.17	1.15	6.13	1.04	6.70	1.13	0.871			
RBC 10^{12}/l	WB	4.64	0.28	4.67	0.25	4.96	0.33	0.070			
PLT 10^9/l	PRP	349.00	91.00	347.50	65.00	328.00	44.50	0.236			
PCT ml/l	PRP	0.31	0.07	0.29	0.06	0.28	0.04	0.212			
MPV fl	PRP	8.60	0.50	8.55	0.40	8.50	0.50	0.472			
PDW fl	PRP	14.60	0.20	14.60	0.25	14.50	0.20	0.778			

**Parameter**	**Source**	**Genotypes of rs3756312**						**P value**			
		**AA**		**AG**		**GG**					
		**Median**	**±QD**	**Median**	**±QD**	**Median**	**±QD**	**Kruskal-Wallis test**	**AA vs AG**	**AA vs GG**	**AG vs GG**
PLT 10^9/l	WB	253.00	37.50	229.00	54.00	228.00	40.50	0.121			
PCT ml/l	WB	2.33	0.37	2.08	0.30	2.38	0.30	0.039	0.036	1.000	0.538
MPV fl	WB	9.10	0.65	8.90	0.65	10.10	0.50	0.017	1.000	0.026	0.019
PDW fl	WB	16.00	0.20	16.10	0.15	16.20	0.15	0.245			
WBC 10^9/l	WB	6.24	1.13	6.24	1.03	6.36	1.47	0.886			
RBC 10^{12}/l	WB	4.66	0.26	4.54	0.27	5.22	0.26	0.000	1.000	0.001	0.001
PLT 10^9/l	PRP	349.00	89.50	327.00	68.00	344.00	40.25	0.164			
PCT ml/l	PRP	0.31	0.07	0.29	0.07	0.30	0.04	0.156			
MPV fl	PRP	8.65	0.45	8.50	0.43	8.50	0.45	0.542			
PDW fl	PRP	14.60	0.20	14.55	0.25	14.50	0.23	0.741			

Legend: *MPV*, platelet volume; *PCT*, plateletcrit; *PDGFA*, platelet-derived growth factor alpha gene; *PDW*, platelet distribution width; *PLT*, platelets; *PRP*, platelet-rich plasma; *QD*, Quartile Deviation; *WB*, whole blood.
