# Supplementary material for: Polymorphic Variants of the PDGFRB Gene Influence Efficacy of PRP Therapy in Treating Tennis Elbow: A Prospective Cohort Study
Source: J Clin Med. 2022 Oct 28;11(21):6362. doi: 10.3390/jcm11216362 (PMC9657684; doi:10.3390/jcm11216362)
Supplement: Supplementary file 1 [file jcm-11-06362-s001.zip › Table S3.pdf]

Table S3. Whole blood (WB) and platelet-rich plasma (PRP) parameters values in individuals with particular variants of the *PDGFRB* gene polymorphisms in recessive/dominant model.

Parameter	Source	rs4324662				*P.* Mann-Whitney U test
		CC		CT/TT		
		Median	±QD	Median	±QD	
PLT 10^9/l	WB	246.00	39.00	238.00	43.50	0.225
PCT ml/l	WB	2.28	0.39	2.33	0.33	0.390
MPV fl	WB	9.10	0.70	9.40	0.95	0.531
PDW fl	WB	16.10	0.15	16.10	0.15	0.773
WBC 10^9/l	WB	6.24	1.16	6.36	1.25	0.802
RBC 10^{12}/l	WB	4.66	0.27	4.72	0.35	0.408
PLT 10^9/l	PRP	353.00	70.50	327.00	59.75	**0.024**
PCT ml/l	PRP	0.31	0.05	0.27	0.05	**0.009**
MPV fl	PRP	8.80	0.40	8.30	0.32	**0.006**
PDW fl	PRP	14.60	0.20	14.40	0.18	**0.005**

Parameter	Source	rs758588				*P.* Mann-Whitney U test
		AA		AG/GG		
		Median	±QD	Median	±QD	
PLT 10^9/l	WB	221.00	43.50	246.00	35.75	0.234
PCT ml/l	WB	2.13	0.22	2.32	0.39	0.261
MPV fl	WB	9.65	0.85	9.10	0.70	0.687
PDW fl	WB	16.15	0.10	16.05	0.15	0.265
WBC 10^9/l	WB	6.60	1.97	6.24	1.13	0.644
RBC 10^{12}/l	WB	4.63	0.48	4.67	0.29	0.827
PLT 10^9/l	PRP	335.00	44.00	349.00	75.50	0.553
PCT ml/l	PRP	0.29	0.04	0.30	0.06	0.613
MPV fl	PRP	8.50	0.68	8.60	0.40	0.997
PDW fl	PRP	14.60	0.30	14.60	0.25	1.000

Parameter	Source	rs3828610				*P.* Mann-Whitney U test
		CC		AA/AC		
		Median	±QD	Median	±QD	
PLT 10^9/l	WB	228.00	40.50	250.00	36.50	0.211
PCT ml/l	WB	2.33	0.25	2.22	0.39	0.920
MPV fl	WB	9.80	0.55	9.00	0.70	**0.003**
PDW fl	WB	16.20	0.15	16.00	0.15	0.127
WBC 10^9/l	WB	6.70	1.13	6.17	1.11	0.761
RBC 10^{12}/l	WB	4.96	0.33	4.66	0.25	**0.024**
PLT 10^9/l	PRP	328.00	44.50	349.00	71.50	0.301
PCT ml/l	PRP	0.28	0.04	0.31	0.06	0.413
MPV fl	PRP	8.50	0.50	8.60	0.40	0.619
PDW fl	PRP	14.50	0.20	14.60	0.25	0.616

Parameter	Source	rs3756311				*P.* Mann-Whitney U test
		GG		AA/AG		
		Median	±QD	Median	±QD	
PLT 10^9/l	WB	228.00	40.50	250.00	36.50	0.211
PCT ml/l	WB	2.33	0.25	2.22	0.39	0.920
MPV fl	WB	9.80	0.55	9.00	0.70	**0.003**
PDW fl	WB	16.20	0.15	16.00	0.15	0.127
WBC 10^9/l	WB	6.70	1.13	6.17	1.11	0.761
RBC 10^{12}/l	WB	4.96	0.33	4.66	0.25	**0.024**
PLT 10^9/l	PRP	328.00	44.50	349.00	71.50	0.301
PCT ml/l	PRP	0.28	0.04	0.31	0.06	0.413
MPV fl	PRP	8.50	0.50	8.60	0.40	0.619
PDW fl	PRP	14.50	0.20	14.60	0.25	0.616

Parameter	Source	rs3756312				*P.* Mann-Whitney
		GG		AA/AG		

		Median	±QD	Median	±QD	U test
PLT 10^9/l	WB	228.00	40.50	246.00	38.75	0.419
PCT ml/l	WB	2.38	0.30	2.21	0.38	0.677
MPV fl	WB	10.10	0.50	9.00	0.70	**0.005**
PDW fl	WB	16.20	0.15	16.05	0.15	0.183
WBC 10^9/l	WB	6.36	1.47	6.24	1.11	0.677
RBC 10^{12}/l	WB	5.22	0.26	4.65	0.26	**0.000**
PLT 10^9/l	PRP	344.00	40.25	343.00	68.00	0.630
PCT ml/l	PRP	0.30	0.04	0.30	0.06	0.590
MPV fl	PRP	8.50	0.45	8.60	0.40	0.534
PDW fl	PRP	14.50	0.23	14.60	0.25	0.446

Legend: *MPV*, platelet volume; *PCT*, plateletcrit; *PDGFRB*, platelet-derived growth factor receptor beta gene; *PDW*, platelet distribution width; *PLT*, platelets; *PRP*, platelet-rich plasma; *QD*, Quartile Deviation; *WB*, whole blood.
