# Supplementary material for: Polymorphic Variants of the PDGFRB Gene Influence Efficacy of PRP Therapy in Treating Tennis Elbow: A Prospective Cohort Study
Source: J Clin Med. 2022 Oct 28;11(21):6362. doi: 10.3390/jcm11216362 (PMC9657684; doi:10.3390/jcm11216362)
Supplement: Supplementary file 1 [file jcm-11-06362-s001.zip › Table S4.pdf]

Table S4. PROMs values in individuals with particular genotypes of the *PDGFRB* gene polymorphisms in additive model.

PROM	week	Genotypes of rs4324662						*P* value			
		CC		CT		TT					
		Median	±QD	Median	±QD	Median	±QD	Kruskal-Wallis test	CC vs CT	CC vs TT	CT vs TT
VAS	0	6.00	2.00	5.00	1.50	5.00	1.50	0.435			
	2	4.00	1.50	4.00	1.50	2.50	2.00	0.504			
	4	3.00	1.50	3.00	1.50	3.50	3.50	0.986			
	8	3.00	1.50	3.50	2.25	0.00	1.50	0.170			
	12	2.00	1.50	3.00	2.00	0.50	1.75	0.470			
	24	2.00	1.50	3.00	2.50	1.50	2.50	0.764			
	52	1.00	2.00	2.50	2.50	1.50	2.00	0.569			
	104	1.00	1.50	1.00	1.50	0.00	0.50	0.236			
ΔVAS (vs week 0)	2	2.00	1.50	1.00	1.00	1.50	1.00	0.456			
	4	2.75	2.00	1.00	1.25	0.50	2.00	0.286			
	8	3.00	2.50	2.00	1.50	3.00	2.00	0.492			
	12	3.00	2.00	2.50	1.50	3.00	1.75	0.829			
	24	3.00	2.50	2.00	1.50	2.50	1.50	0.426			
	52	4.00	2.50	3.00	2.00	2.50	1.00	0.391			
	104	4.00	3.00	4.00	2.00	4.00	1.00	0.993			
QDASH	0	54.77	11.37	45.45	17.05	52.27	11.36	0.282			
	2	40.91	13.64	36.36	17.61	34.09	20.45	0.914			
	4	34.09	13.64	37.50	14.77	26.14	30.68	0.869			
	8	30.68	16.48	37.50	22.16	0.00	18.18	0.171			
	12	27.27	14.77	34.09	20.45	6.82	21.59	0.466			
	24	25.00	20.45	31.82	20.45	12.50	18.75	0.617			
	52	14.77	20.45	23.86	24.43	26.14	26.14	0.811			
	104	15.91	21.59	9.09	13.64	0.00	2.27	0.101			
ΔQDASH (vs week 0)	2	6.82	12.50	-1.14	11.93	21.59	17.05	0.305			
	4	16.59	15.91	6.81	12.50	31.81	27.27	0.136			
	8	18.18	19.31	7.95	17.04	44.32	14.77	0.027	0.116	0.435	0.089
	12	20.45	17.71	15.91	17.61	37.50	18.18	0.141			
	24	23.64	20.39	15.91	17.04	52.27	17.61	0.168			
	52	27.27	19.32	20.45	17.04	40.90	22.73	0.147			
	104	31.82	23.80	27.27	15.90	52.27	9.09	0.144			
PRTEE	0	53.00	13.13	45.50	16.38	53.00	12.50	0.475			

	2	30.00	14.25	28.75	17.38	30.50	25.63	0.951
	4	27.50	13.50	23.25	12.75	30.00	30.38	0.910
	8	22.00	14.25	24.00	19.38	0.00	13.75	0.156
	12	19.50	14.38	21.25	15.13	2.25	16.00	0.353
	24	14.50	17.25	16.00	15.00	19.25	22.25	0.946
	52	11.25	14.00	15.00	17.50	10.00	15.88	0.834
	104	9.00	14.00	5.00	8.25	0.00	2.50	0.129
ΔPRTEE (vs week 0)	2	17.50	13.75	14.00	7.13	6.75	13.13	0.480
	4	26.50	15.75	17.75	10.75	14.75	17.88	0.282
	8	29.50	18.50	23.00	13.00	36.75	17.50	0.106
	12	29.50	18.25	26.75	14.63	35.00	17.50	0.346
	24	33.50	20.75	27.40	14.00	26.25	15.00	0.492
	52	36.00	19.75	27.75	12.38	35.50	17.88	0.421
	104	38.00	17.38	37.00	13.25	53.00	10.00	0.510

**PROM**	**week**	**Genotypes of rs758588**						***P* value**			
		**AA**		**AG**		**GG**					
		**Median**	**±QD**	**Median**	**±QD**	**Median**	**±QD**	**Kruskal-Wallis test**	**AA vs AG**	**AA vs GG**	**AG vs GG**
VAS	0	5.00	1.00	6.00	1.50	6.00	2.00	0.383			
	2	4.00	1.00	4.00	2.00	3.50	1.50	0.552			
	4	2.00	1.50	3.00	1.50	3.00	1.50	0.277			
	8	1.00	1.50	4.00	2.50	3.00	1.50	0.043	0.040	0.104	1.000
	12	1.00	1.00	4.00	2.00	2.00	1.50	0.333			
	24	0.00	1.50	3.00	2.00	2.00	1.75	0.025	0.023	0.143	0.472
	52	1.00	2.00	3.00	2.50	1.00	2.00	0.468			
	104	0.00	1.00	1.00	1.50	1.00	1.50	0.166			
ΔVAS (vs week 0)	2	1.00	2.00	1.00	1.00	2.00	1.50	0.430			
	4	2.00	1.50	1.00	1.50	3.00	2.00	0.284			
	8	3.00	2.00	2.00	1.50	3.00	2.50	0.313			
	12	4.00	2.00	2.50	1.50	3.00	2.00	0.544			
	24	4.00	1.50	2.00	1.50	3.00	2.50	0.228			
	52	3.00	2.00	3.00	2.00	4.00	2.50	0.480			
	104	4.00	1.50	4.00	2.00	4.00	3.00	0.979			
QDASH	0	34.09	12.50	51.14	13.64	54.54	11.37	0.188			
	2	25.00	13.64	43.18	17.05	40.91	13.64	0.601			
	4	36.36	19.32	38.64	14.77	34.09	13.64	0.671			

	8	18.18	30.68	40.91	21.59	31.82	17.05	0.348			
	12	27.27	28.38	35.23	18.18	27.27	15.91	0.840			
	24	2.27	19.32	31.82	19.32	22.73	20.45	0.199			
	52	22.73	26.14	23.86	23.86	15.91	20.45	0.888			
	104	0.00	10.23	9.09	11.37	17.50	21.59	0.237			
ΔQDASH (vs week 0)	2	2.27	20.45	-1.14	12.50	6.82	12.50	0.444			
	4	9.08	20.46	6.81	12.50	17.27	15.91	0.295			
	8	15.91	22.73	7.95	18.18	18.18	19.31	0.364			
	12	18.17	14.77	15.91	19.32	20.45	17.18	0.657			
	24	27.27	29.55	15.91	18.18	22.27	21.46	0.536			
	52	20.45	14.77	22.73	19.32	25.00	18.82	0.292			
	104	27.27	18.18	31.81	15.91	31.81	23.87	0.899			
PRTEE	0	35.50	15.75	52.75	14.75	53.00	12.50	0.144			
	2	20.00	13.25	37.25	18.50	29.50	14.00	0.300			
	4	20.00	10.50	24.75	16.00	27.50	13.25	0.299			
	8	7.50	12.00	29.00	19.00	22.00	14.50	0.043	0.037	0.104	1.000
	12	15.00	9.00	26.25	17.50	20.00	14.75	0.227			
	24	4.00	15.75	21.50	14.25	14.00	17.75	0.131			
	52	15.00	9.50	16.00	21.25	11.50	14.00	0.562			
	104	0.00	7.25	5.00	8.25	9.25	14.38	0.191			
ΔPRTEE (vs week 0)	2	11.00	9.00	14.25	7.00	17.50	13.75	0.447			
	4	12.00	11.75	18.25	11.50	26.50	15.75	0.281			
	8	23.50	13.50	23.50	13.00	29.25	18.00	0.366			
	12	21.00	18.00	27.00	16.25	29.50	18.00	0.695			
	24	27.00	9.00	27.50	14.00	32.50	20.75	0.651			
	52	21.00	17.25	30.50	12.70	35.50	19.63	0.637			
	104	30.50	18.00	38.50	12.25	38.00	17.50	0.705			
		5.00	1.00	6.00	1.50	6.00	2.00				

**PROM**	**week**	**Genotypes of rs3828610**						***P* value**			
		**AA**		**AC**		**CC**					
		**Median**	**±QD**	**Median**	**±QD**	**Median**	**±QD**	**Kruskal-Wallis test**	**AA vs AC**	**AA vs CC**	**AC vs CC**
VAS	0	6.00	1.88	6.00	1.50	6.00	1.50	0.918			
	2	3.00	1.50	4.00	1.50	3.00	2.00	0.294			
	4	3.00	2.00	3.00	1.50	3.00	1.00	0.823			
	8	3.00	1.50	3.00	2.00	2.00	2.00	0.406			
	12	2.00	1.50	3.00	2.00	1.00	2.00	0.251			

	24	2.00	2.00	2.50	2.00	1.00	1.50	0.320			
	52	1.50	2.00	2.00	2.50	1.00	1.50	0.441			
	104	1.00	2.00	1.00	1.50	0.00	1.00	0.441			
ΔVAS	2	1.00	1.50	1.00	1.50	2.00	1.00	0.573			
(vs week 0)	4	2.00	2.00	2.00	1.50	3.00	1.50	0.649			
	8	2.00	2.00	2.00	1.50	3.00	2.50	0.353			
	12	2.00	1.75	2.00	2.00	4.00	1.50	0.292			
	24	2.00	1.50	3.00	2.00	4.00	1.50	0.354			
	52	2.00	2.50	3.50	2.50	4.00	2.00	0.572			
	104	3.00	2.50	4.00	2.25	4.00	2.00	0.410			
QDASH	0	51.14	11.87	47.72	14.77	59.09	11.37	0.282			
	2	38.64	12.50	40.91	18.18	36.36	18.18	0.753			
	4	34.09	11.36	36.36	17.05	38.64	14.77	0.853			
	8	30.68	15.91	36.36	20.45	27.27	26.14	0.733			
	12	27.27	13.07	29.55	22.73	34.09	18.18	0.857			
	24	29.55	19.32	28.41	23.86	20.45	13.64	0.072			
	52	14.77	20.45	22.73	25.00	18.18	25.00	0.964			
	104	12.50	23.86	15.91	13.64	4.55	10.23	0.347			
ΔQDASH	2	6.81	11.47	-2.28	12.50	13.64	14.77	0.078			
(vs week 0)	4	15.90	15.77	7.95	11.32	22.72	20.45	0.396			
	8	13.63	15.47	7.95	17.04	34.09	15.91	0.078			
	12	19.31	14.21	13.63	15.91	30.91	15.91	0.105			
	24	20.45	19.32	15.91	14.78	38.64	13.64	0.008	1.000	0.014	0.015
	52	18.04	19.32	19.31	14.77	36.36	17.04	0.184			
	104	29.54	25.00	27.27	15.34	46.81	7.96	0.039	1.000	0.060	0.060
PRTEE	0	50.75	12.00	55.00	14.25	52.50	14.50	0.531			
	2	28.00	14.25	34.00	16.75	24.00	19.00	0.778			
	4	25.50	12.00	25.00	17.50	20.50	14.25	0.932			
	8	23.25	12.63	27.00	20.00	19.50	13.75	0.483			
	12	19.50	11.25	22.00	19.00	19.50	17.75	0.495			
	24	19.50	15.75	17.00	18.25	9.00	10.75	0.088			
	52	11.00	15.50	14.00	15.50	11.50	9.00	0.456			
	104	8.50	15.50	8.00	12.50	1.50	5.50	0.190			
ΔPRTEE	2	13.00	14.50	16.75	10.75	15.50	8.50	0.836			
(vs week 0)	4	21.00	13.00	21.50	13.00	24.00	13.25	0.861			
	8	24.75	17.00	25.00	14.00	34.50	19.75	0.362			
	12	26.00	16.00	27.00	16.00	34.00	15.00	0.487			

	24	25.00	16.00	27.50	14.50	38.00	12.75	0.059			
	52	30.75	19.75	32.25	13.25	40.00	15.50	0.246			
	104	37.50	19.75	37.25	12.50	46.00	16.00	0.157			

**PROM**	**week**	**Genotypes of rs3756311**						***P* value**			
		**AA**		**AG**		**GG**					
		**Median**	**±QD**	**Median**	**±QD**	**Median**	**±QD**	**Kruskal-Wallis test**	**AA vs AG**	**AA vs GG**	**AG vs GG**
VAS	0	6.00	2.00	6.00	1.50	6.00	1.50	0.941			
	2	3.50	1.50	4.00	1.25	3.00	2.00	0.323			
	4	3.00	2.00	3.00	1.50	3.00	1.00	0.802			
	8	3.00	1.50	3.00	2.00	2.00	2.00	0.406			
	12	2.00	1.50	3.00	2.00	1.00	2.00	0.246			
	24	2.00	2.00	3.00	2.00	1.00	1.50	0.323			
	52	1.00	2.00	2.50	2.50	1.00	1.50	0.424			
	104	1.00	1.50	1.00	1.50	0.00	1.00	0.448			
ΔVAS (vs week 0)	2	1.00	1.50	1.00	1.50	2.00	1.00	0.571			
	4	2.00	2.00	2.00	1.50	3.00	1.50	0.694			
	8	2.00	2.00	2.00	1.50	3.00	2.50	0.358			
	12	2.00	2.00	2.00	2.00	4.00	1.50	0.293			
	24	2.00	1.50	2.50	2.00	4.00	1.50	0.364			
	52	2.00	2.50	3.00	2.50	4.00	2.00	0.585			
	104	3.00	2.75	4.00	2.00	4.00	2.00	0.524			
QDASH	0	52.27	11.37	46.59	15.34	59.09	11.37	0.252			
	2	38.64	12.50	40.91	17.22	36.36	18.18	0.720			
	4	34.09	11.36	36.36	17.05	38.64	14.77	0.843			
	8	29.55	15.91	36.36	20.45	27.27	26.14	0.719			
	12	27.27	12.50	30.68	22.16	34.09	18.18	0.805			
	24	29.55	20.45	31.82	23.86	20.45	13.64	0.079			
	52	13.64	20.45	23.86	24.43	18.18	25.00	0.971			
	104	11.36	21.59	17.05	20.46	4.55	10.23	0.333			
ΔQDASH (vs week 0)	2	6.81	11.47	-2.28	12.50	13.64	14.77	0.052			
	4	15.90	15.77	6.82	12.45	22.72	20.45	0.288			
	8	13.63	15.91	6.82	17.04	34.09	15.91	0.057			
	12	20.45	13.64	13.63	17.05	30.91	15.91	0.074			
	24	20.45	19.32	15.91	15.91	38.64	13.64	0.008	1.000	0.020	0.010
	52	18.17	18.18	18.18	15.91	36.36	17.04	0.155			
	104	29.55	23.87	27.27	15.91	46.81	7.96	0.036	1.000	0.086	0.041

PRTEE	0	51.00	12.50	54.50	14.00	52.50	14.50	0.612			
	2	28.50	14.25	33.75	16.38	24.00	19.00	0.824			
	4	25.00	12.00	25.25	17.25	20.50	14.25	0.931			
	8	22.00	15.25	26.75	19.00	19.50	13.75	0.480			
	12	19.00	11.50	22.50	17.88	19.50	17.75	0.475			
	24	18.25	16.63	18.00	18.25	9.00	10.75	0.092			
	52	11.00	15.50	14.50	15.38	11.50	9.00	0.451			
	104	7.50	15.00	8.50	14.00	1.50	5.50	0.189			
ΔPRTEE (vs week 0)	2	12.25	14.50	17.00	10.75	15.50	8.50	0.801			
	4	21.25	13.00	21.50	13.00	24.00	13.25	0.894			
	8	25.00	17.75	24.50	14.00	34.50	19.75	0.366			
	12	28.00	17.00	27.00	16.00	34.00	15.00	0.460			
	24	26.50	17.75	27.45	14.50	38.00	12.75	0.063			
	52	32.00	19.75	32.00	14.00	40.00	15.50	0.275			
	104	37.50	19.75	37.00	12.25	46.00	16.00	0.196			
**PROM**	**week**	**Genotypes of rs3756312**						***P* value**			
		**AA**		**AG**		**GG**					
		**Median**	**±QD**	**Median**	**±QD**	**Median**	**±QD**	**Kruskal-Wallis test**	**AA vs AG**	**AA vs GG**	**AG vs GG**
VAS	0	6.00	2.00	6.00	1.50	5.50	1.75	0.858			
	2	3.00	1.50	4.00	1.50	3.00	1.75	0.087			
	4	3.00	2.00	3.00	1.50	3.00	2.00	0.883			
	8	3.00	1.50	3.00	2.00	2.50	2.50	0.707			
	12	2.00	1.50	3.00	2.00	1.50	2.50	0.723			
	24	2.00	1.50	2.00	2.00	2.50	2.50	0.985			
	52	1.00	2.00	2.00	2.50	2.50	2.00	0.830			
	104	1.00	2.00	1.00	1.00	0.50	1.25	0.673			
ΔVAS (vs week 0)	2	2.00	2.00	1.00	1.50	2.00	1.00	0.363			
	4	2.00	2.00	2.00	1.50	2.50	2.25	0.960			
	8	3.00	2.00	2.00	1.50	3.00	2.50	0.975			
	12	3.00	2.25	3.00	2.00	3.00	2.25	0.926			
	24	3.00	2.00	3.00	2.00	2.50	1.50	0.766			
	52	4.00	2.50	3.00	2.50	3.00	1.75	0.814			
	104	3.00	3.00	4.00	2.00	4.00	2.25	0.686			
QDASH	0	52.27	11.93	45.45	15.34	57.95	6.82	0.454			
	2	40.91	12.50	42.05	17.05	32.95	13.07	0.334			
	4	34.09	11.36	36.36	16.48	30.68	17.05	0.903			

	8	31.82	16.48	36.36	19.32	20.45	30.68	0.511
	12	27.27	13.07	34.09	21.02	15.91	24.43	0.452
	24	25.00	20.45	31.82	22.73	20.45	13.07	0.206
	52	13.64	20.45	23.86	21.59	18.18	25.57	0.500
	104	13.64	21.59	15.91	14.78	3.41	10.23	0.173
ΔQDASH	2	6.81	12.50	2.18	12.50	19.32	18.75	0.055
(vs week 0)	4	15.90	15.77	9.09	12.50	27.27	26.70	0.359
	8	13.63	16.03	11.36	18.18	36.36	26.14	0.132
	12	20.45	14.78	15.97	16.59	31.82	26.71	0.246
	24	22.27	19.32	15.91	18.18	45.45	22.16	0.057
	52	21.59	18.19	15.91	15.91	40.91	20.46	0.160
	104	29.55	23.87	27.27	12.50	52.27	8.52	0.051
PRTEE	0	51.25	12.88	53.75	14.63	53.00	12.63	0.908
	2	28.00	14.25	33.75	16.50	23.25	18.38	0.533
	4	25.50	12.25	25.25	14.25	19.00	17.00	0.695
	8	21.75	12.50	25.50	17.25	19.75	27.63	0.746
	12	17.75	11.38	21.75	15.38	8.25	25.75	0.518
	24	14.50	16.25	16.00	18.00	11.75	16.25	0.501
	52	7.50	13.50	14.50	15.00	11.25	16.00	0.616
	104	8.25	15.00	8.00	10.50	2.75	4.63	0.184
ΔPRTEE	2	15.00	14.25	14.50	7.75	14.50	12.50	0.887
(vs week 0)	4	21.50	15.00	21.50	12.75	25.75	16.88	0.874
	8	28.00	17.13	24.50	14.00	35.25	26.00	0.804
	12	29.25	17.13	27.00	14.25	28.50	26.38	0.865
	24	30.50	19.50	27.45	14.00	36.00	18.38	0.562
	52	33.25	20.00	29.00	12.50	40.50	18.38	0.876
	104	37.75	20.00	37.50	13.50	49.25	18.13	0.348

Legend: *PDGFRB*, gene for platelet-derived growth factor beta receptor; QD, Quartile Deviation; PROMs, patient-reported outcome measures; VAS, Visual Analog Scale; QDASH, quick version of Disabilities of the Arm, Shoulder and Hand score; PRTEE, Patient-Rated Tennis Elbow Evaluation.
