# Supplementary material for: Polymorphic Variants of the PDGFRB Gene Influence Efficacy of PRP Therapy in Treating Tennis Elbow: A Prospective Cohort Study
Source: J Clin Med. 2022 Oct 28;11(21):6362. doi: 10.3390/jcm11216362 (PMC9657684; doi:10.3390/jcm11216362)
Supplement: Supplementary file 1 [file jcm-11-06362-s001.zip › Table S5.pdf]

Table S5. PROMs values in TT homozygotes and carriers of the C allele of the rs4324662 *PDGFRB* gene polymorphism.

PROMs		TT rs4324662		CT+CC rs4324662		*p* Mann-Whitney U test
	week	median	± QD	median	± QD	
VAS	0	5.00	1.50	6.00	1.88	0.329
	2	2.50	2.00	4.00	1.50	0.273
	4	3.50	3.50	3.00	1.50	0.877
	8	0.00	1.50	3.00	2.00	0.067
	12	0.50	1.75	3.00	1.50	0.227
	24	1.50	2.50	2.00	2.00	0.652
	52	1.50	2.00	2.00	2.00	0.674
	104	0.00	0.50	1.00	1.50	0.179
ΔVAS (vs week 0)	2	1.50	1.00	1.00	1.50	0.595
	4	0.50	2.00	2.00	2.00	0.341
	8	3.00	2.00	2.00	2.00	0.600
	12	3.00	1.75	3.00	2.00	0.973
	24	2.50	1.50	3.00	2.00	0.574
	52	2.50	1.00	4.00	2.00	0.637
	104	4.00	1.00	4.00	2.00	1.000
QDASH	0	52.27	11.36	52.27	13.64	0.715
	2	34.09	20.45	38.64	15.91	0.773
	4	26.14	30.68	36.36	13.64	0.668
	8	0.00	18.18	34.09	18.75	0.087
	12	6.82	21.59	29.55	17.05	0.237
	24	12.50	18.75	25.00	21.59	0.329
	52	26.14	26.14	18.18	22.73	0.855
	104	0.00	2.28	14.77	21.59	0.050
ΔQDASH (vs week 0)	2	21.59	17.05	6.81	13.63	0.450
	4	31.81	27.27	12.50	15.91	0.415
	8	44.32	14.77	15.90	18.18	0.088
	12	37.50	18.18	18.18	17.05	0.211
	24	52.27	17.61	20.45	18.18	0.219
	52	40.90	22.73	20.45	20.45	0.659
	104	52.27	9.09	29.54	22.73	0.064
PRTEE	0	53.00	12.50	51.75	13.75	0.900
	2	30.50	25.63	29.50	16.00	0.915
	4	30.00	30.38	24.50	13.25	0.763
	8	0.00	13.75	23.00	15.63	0.064
	12	2.25	16.00	20.50	14.63	0.157
	24	19.25	22.25	14.75	16.75	0.747
	52	10.00	15.88	11.75	15.25	0.597
	104	0.00	2.50	7.50	13.38	0.097
ΔPRTEE (vs week 0)	2	6.75	13.13	15.25	11.50	0.509
	4	14.75	17.88	21.50	13.50	0.647
	8	36.75	17.50	25.50	16.75	0.303
	12	35.00	17.50	29.00	17.00	0.521
	24	26.25	15.00	30.50	19.00	0.804
	52	35.50	17.88	33.00	17.25	0.903
	104	53.00	10.00	38.00	16.75	0.301

Legend: QD, Quartile Deviation; VAS, Visual Analog Scale; QDASH, quick version of Disabilities of the Arm, Shoulder and Hand score; PROM, Patient-Reported Outcome Measures; PRTEE, Patient-Rated Tennis Elbow Evaluation.
