# Supplementary material for: Polymorphic Variants of the PDGFRB Gene Influence Efficacy of PRP Therapy in Treating Tennis Elbow: A Prospective Cohort Study
Source: J Clin Med. 2022 Oct 28;11(21):6362. doi: 10.3390/jcm11216362 (PMC9657684; doi:10.3390/jcm11216362)
Supplement: Supplementary file 1 [file jcm-11-06362-s001.zip › Table S6.pdf]

Table S6. PROMs values in AA homozygotes and carriers of the G allele of the rs758588 *PDGFRB* gene polymorphism.

**PROMs**		**AA rs758588**		**AG+GG rs758588**		***p***
	week	median	± QD	median	± QD	**Mann-Whitney U test**
VAS	0	5.00	1.00	6.00	2.00	0.201
	2	4.00	1.00	4.00	1.50	0.433
	4	2.00	1.50	3.00	1.50	0.157
	8	1.00	1.50	3.00	2.00	**0.020**
	12	1.00	1.00	3.00	1.50	0.152
	24	0.00	1.50	2.00	2.00	**0.022**
	52	1.00	2.00	2.00	2.00	0.478
	104	0.00	1.00	1.00	1.50	0.075
ΔVAS (vs week 0)	2	1.00	2.00	1.00	1.50	0.902
	4	2.00	1.50	2.00	2.00	0.950
	8	3.00	2.00	2.00	2.00	0.381
	12	4.00	2.00	3.00	2.00	0.445
	24	4.00	1.50	3.00	2.00	0.472
	52	3.00	2.00	4.00	2.50	0.872
	104	4.00	1.50	4.00	2.50	0.847
QDASH	0	34.09	12.50	52.27	11.37	0.080
	2	25.00	13.64	40.91	15.91	0.344
	4	36.36	19.32	36.36	13.64	0.569
	8	18.18	30.68	34.09	18.18	0.239
	12	27.27	28.38	29.55	17.05	0.581
	24	2.27	19.32	25.00	20.46	0.085
	52	22.73	26.14	18.18	22.73	0.976
	104	0.00	10.23	15.91	21.59	0.142
ΔQDASH (vs week 0)	2	2.27	20.45	6.81	13.63	0.601
	4	9.08	20.46	13.63	14.77	0.371
	8	15.91	22.73	15.90	18.24	0.813
	12	18.17	14.77	19.31	17.05	0.675
	24	27.27	29.55	20.45	19.25	0.820
	52	20.45	14.77	22.73	19.32	0.269
	104	27.27	18.18	31.81	22.73	0.723
PRTEE	0	35.50	15.75	53.00	13.75	0.038
	2	20.00	13.25	31.00	16.38	0.150
	4	20.00	10.50	25.50	14.13	0.142
	8	7.50	12.00	24.50	15.75	**0.021**
	12	15.00	9.00	21.50	15.00	0.101
	24	4.00	15.75	15.50	16.50	0.061
	52	15.00	9.50	11.50	15.75	0.338
	104	0.00	7.25	7.50	14.00	14.00
ΔPRTEE (vs week 0)	2	11.00	9.00	15.50	12.50	0.321
	4	12.00	11.75	22.00	13.75	0.342
	8	23.50	13.50	27.75	17.00	0.898
	12	21.00	18.00	29.00	17.25	0.964
	24	27.00	9.00	31.00	19.25	0.665
	52	21.00	17.25	33.50	18.25	0.513
	104	30.50	18.00	38.25	16.00	0.470

Legend: QD, Quartile Deviation; VAS, Visual Analog Scale; QDASH, quick version of Disabilities of the Arm, Shoulder and Hand score; PROM, Patient-Reported Outcome Measures; PRTEE, Patient-Rated Tennis Elbow Evaluation.
