# Supplementary material for: Polymorphic Variants of the PDGFRB Gene Influence Efficacy of PRP Therapy in Treating Tennis Elbow: A Prospective Cohort Study
Source: J Clin Med. 2022 Oct 28;11(21):6362. doi: 10.3390/jcm11216362 (PMC9657684; doi:10.3390/jcm11216362)
Supplement: Supplementary file 1 [file jcm-11-06362-s001.zip › Table S7.pdf]

Table S7. PROMs values in CC homozygotes and A allele carriers of the rs3828610 *PDGFRB* gene polymorphism.

**PROMs**		**CC rs3828610**		**AC+AA rs3828610**		***P***
	week	median	± QD	median	± QD	**Mann-Whitney U test**
VAS	0	6.00	1.50	6.00	1.75	0.909
	2	3.00	2.00	4.00	1.50	0.268
	4	3.00	1.00	3.00	1.50	0.570
	8	2.00	2.00	3.00	2.00	0.181
	12	1.00	2.00	3.00	2.00	0.137
	24	1.00	1.50	2.00	2.00	0.136
	52	1.00	1.50	2.00	2.50	0.233
	104	0.00	1.00	1.00	1.50	0.206
ΔVAS (vs week 0)	2	2.00	1.00	1.00	1.50	0.293
	4	3.00	1.50	2.00	2.00	0.514
	8	3.00	2.50	2.00	2.00	0.191
	12	4.00	1.50	2.00	2.00	0.118
	24	4.00	1.50	2.00	2.00	0.187
	52	4.00	2.00	3.00	2.00	0.336
	104	4.00	2.00	4.00	2.00	0.562
QDASH	0	59.09	11.37	50.00	13.64	0.118
	2	36.36	18.18	39.77	14.77	0.833
	4	38.64	14.77	36.36	13.64	0.580
	8	27.27	26.14	34.09	18.18	0.433
	12	34.09	18.18	27.27	17.05	0.659
	24	20.45	13.64	29.55	21.59	**0.034**
	52	18.18	25.00	18.18	22.73	0.826
	104	4.55	10.23	15.91	17.09	0.146
ΔQDASH (vs week 0)	2	13.64	14.77	4.54	11.37	0.087
	4	22.72	20.45	11.35	13.64	0.274
	8	34.09	15.91	11.36	16.02	**0.038**
	12	30.91	15.91	17.04	17.05	0.063
	24	38.64	13.64	15.91	19.31	**0.002**
	52	36.36	17.04	18.18	19.32	0.073
	104	46.81	6.96	28.36	20.09	**0.011**
PRTEE	0	52.50	14.50	52.00	13.25	0.938
	2	24.00	19.00	30.25	14.50	0.788
	4	20.50	14.25	25.25	13.00	0.710
	8	19.50	13.75	24.50	16.00	0.237
	12	19.50	17.75	20.00	15.00	0.288
	24	9.00	10.75	18.00	18.00	**0.035**
	52	11.50	9.00	12.00	15.75	0.211
	104	1.50	5.50	8.00	14.75	0.069
ΔPRTEE (vs week 0)	2	15.50	8.50	14.50	13.00	0.699
	4	24.00	13.25	21.50	13.00	0.712
	8	34.50	19.75	24.75	15.50	0.157
	12	34.00	15.00	27.00	16.50	0.233
	24	38.00	12.75	27.45	15.75	**0.023**
	52	40.00	15.50	32.00	16.88	0.154
	104	46.00	16.00	37.50	15.75	0.090

Legend: QD, Quartile Deviation; VAS, Visual Analog Scale; QDASH, quick version of Disabilities of the Arm, Shoulder and Hand score; PROM, Patient-Reported Outcome Measures; PRTEE, Patient-Rated Tennis Elbow Evaluation.
