# Supplementary material for: Polymorphic Variants of the PDGFRB Gene Influence Efficacy of PRP Therapy in Treating Tennis Elbow: A Prospective Cohort Study
Source: J Clin Med. 2022 Oct 28;11(21):6362. doi: 10.3390/jcm11216362 (PMC9657684; doi:10.3390/jcm11216362)
Supplement: Supplementary file 1 [file jcm-11-06362-s001.zip › Table S9.pdf]

Table S9. PROMs values in GG homozygotes and A allele carriers of the rs3756312 *PDGFRB* gene polymorphism.

PROMs		GG rs3756312		AG+AA rs3756312		*P*
	week	median	± QD	median	± QD	Mann-Whitney U test
VAS	0	5.50	1.75	6.00	1.63	0.635
	2	3.00	1.75	4.00	1.50	0.089
	4	3.00	2.00	3.00	1.50	0.665
	8	2.50	2.50	3.00	2.00	0.407
	12	1.50	2.50	3.00	1.50	0.479
	24	2.50	2.50	2.00	2.00	0.983
	52	2.50	2.00	1.50	2.00	0.873
	104	0.50	1.25	1.00	1.50	0.477
ΔVAS (vs week 0)	2	2.00	1.00	1.00	1.50	0.309
	4	2.50	2.25	2.00	1.50	0.867
	8	3.00	2.50	2.00	2.00	0.829
	12	3.00	2.25	3.00	2.00	0.807
	24	2.50	1.50	3.00	2.00	0.513
	52	3.00	1.75	4.00	2.50	0.570
	104	4.00	2.20	4.00	2.00	0.822
QDASH	0	57.95	6.82	50.00	13.64	0.233
	2	32.95	13.07	40.91	15.91	0.205
	4	30.68	17.05	36.36	14.77	0.792
	8	20.45	30.68	34.09	17.61	0.253
	12	15.91	24.43	29.55	17.05	0.335
	24	20.45	13.07	28.41	21.02	0.076
	52	18.18	25.57	18.18	21.59	0.505
	104	3.41	10.23	15.91	21.59	0.063
ΔQDASH (vs week 0)	2	19.32	18.75	4.55	11.37	**0.046**
	4	27.27	26.70	11.36	13.64	0.258
	8	36.36	26.14	13.63	16.02	0.063
	12	31.82	26.71	18.18	17.05	0.207
	24	45.45	22.16	18.17	18.18	**0.025**
	52	40.91	20.46	20.45	19.32	0.151
	104	52.27	8.52	29.54	21.59	**0.015**
PRTEE	0	53.00	12.63	51.75	14.38	0.933
	2	23.25	18.38	30.00	16.00	0.437
	4	19.00	17.00	25.50	13.00	0.456
	8	19.75	27.63	24.00	15.00	0.492
	12	8.25	25.75	20.50	14.00	0.377
	24	11.75	16.25	15.25	16.75	0.249
	52	11.25	16.00	11.75	15.25	0.452
	104	2.75	4.63	8.00	14.00	0.073
ΔPRTEE (vs week 0)	2	14.50	12.50	14.75	11.50	0.686
	4	25.75	16.88	21.50	13.00	0.678
	8	35.25	26.00	25.50	15.50	0.567
	12	28.50	26.38	29.00	15.50	0.784
	24	36.00	18.38	28.50	18.25	0.321
	52	40.50	18.38	32.00	17.00	0.640
	104	49.25	18.13	37.50	15.75	0.189

Legend: QD, Quartile Deviation; VAS, Visual Analog Scale; QDASH, quick version of Disabilities of the Arm, Shoulder and Hand score; PROM, Patient-Reported Outcome Measures; PRTEE, Patient-Rated Tennis Elbow Evaluation.
